# Severe Eczema Herpeticum With Ocular Involvement Leading to Orbital Cellulitis: A Pediatric Case Report

**DOI:** 10.1155/crdm/2155873

**Published:** 2026-06-18

**Authors:** Chu Han Chew, Mohamad Shahidatul-Adha, Ismail Abdul-Salim, Ee Ling Ang

**Affiliations:** ^1^ Department of Ophthalmology, Hospital Pulau Pinang, Jalan Residensi, Georgetown, Penang, Malaysia, moh.gov.my; ^2^ Department of Ophthalmology and Visual Science, School of Medical Sciences, Health Campus, Universiti Sains Malaysia, Kubang Kerian, Kota Bharu, Kelantan, Malaysia, usm.my

**Keywords:** acyclovir, atopic dermatitis, eczema herpeticum, herpes zoster ophthalmicus, punched-out erosion

## Abstract

Eczema herpeticum (EH) is a superimposed cutaneous viral infection that occurs in the context of preexisting dermatoses, most commonly atopic dermatitis (AD). It typically involves the face, neck, and upper trunk. Although ocular involvement is relatively uncommon, it may present diagnostic and therapeutic challenges and pose a significant risk to vision. We report a pediatric case of EH with severe ocular involvement in a young child with underlying AD. Despite early oral antiviral and antibiotic therapy, the condition progressed rapidly to orbital cellulitis. Clinical improvement was observed following escalation to intravenous antiviral therapy, addition of topical antiviral treatment, and broadening of antibiotic coverage, with subsequent full recovery. This case highlights the importance of early recognition, close monitoring, and timely escalation of treatment in EH with ocular involvement to prevent sight‐threatening complications.

## 1. Introduction

Eczema herpeticum (EH), also known as Kaposi varicelliform eruption, is a potentially serious disseminated cutaneous infection caused by herpes simplex virus (HSV), most commonly occurring in patients with underlying atopic dermatitis (AD) [1]. It is characterized by erythematous, monomorphic vesiculopustular eruptions that rapidly evolve into crusted lesions with characteristic “punched‐out” erosions [1]. EH is often accompanied by fever, malaise, and lymphadenopathy, and if not recognized and treated promptly, it may lead to extensive secondary infection and systemic complications, resulting in significant morbidity and mortality [2]. Ocular involvement in EH is uncommon but clinically significant, as it may lead to keratitis with potential risk of vision loss due to corneal scarring [3, 4].

Systemic antiviral therapy remains the cornerstone of treatment, and early initiation is associated with improved outcomes [1, 2]. Although antibiotics are not routinely indicated, they may be required when secondary bacterial infection is suspected, particularly in cases with extensive disease or clinical deterioration despite appropriate antiviral therapy [1, 2].

We report a case of EH with severe ocular involvement in a young child with underlying AD, initially presenting with features suggestive of herpes zoster ophthalmicus (HZO) and subsequently complicated by orbital cellulitis. This case is notable for its aggressive clinical course, diagnostic challenges in the early stage, and progression despite initial systemic antiviral therapy. It highlights the importance of recognizing evolving clinical features, maintaining a high index of suspicion for EH among its mimickers, and the role of timely escalation and adjunctive therapy in preventing sight‐threatening complications.

## 2. Case Presentation

A three‐year‐old Malay boy with underlying AD since infancy presented with a 4‐day history of left eye redness and periorbital swelling. His AD had been intermittently active, predominantly involving the periorbital, facial and flexural areas, and was managed with regular emollients and intermittent use of topical corticosteroids (hydrocortisone 1% cream) during flares. There was no prior history of systemic immunosuppression or other atopic conditions such as asthma or allergic rhinitis. Family history was notable for atopy in the father and paternal relatives.

The illness began with fever for the first 2 days, followed by the onset of left periorbital redness and swelling. The patient was initially seen by a general practitioner on Day 3 of illness, where he was prescribed oral antihistamines and a short course of oral corticosteroids for a presumed allergic reaction or flare of AD. At this stage, no vesicular lesions were noted. He was also treated empirically for presumed preseptal cellulitis with oral antibiotics. However, there was no clinical improvement, and cutaneous vesicular lesions subsequently developed, involving the left periorbital and facial regions, with a few over the neck. He was brought to the eye clinic on Day 6 of illness due to worsening painful and pruritic lesions.

Initial examination revealed multiple monomorphic papulovesicular rashes with early punched‐out erosions, particularly over the left periorbital region, associated with mild eyelid erythema and thick mucoid discharge (Figure 1A). Hutchinson’s sign was negative. Based on the apparent dermatomal distribution, predominantly involving the ophthalmic division of the trigeminal nerve (V1), HZO was initially considered, and oral acyclovir 200 mg five times daily was commenced as first‐line therapy.

**FIGURE 1:**
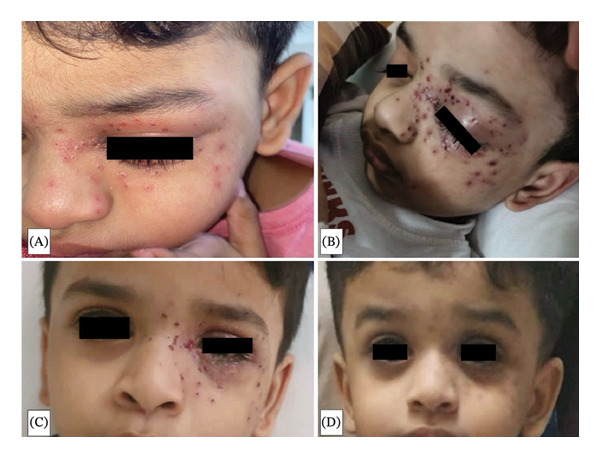
Clinical course of eczema herpeticum with ocular involvement. (A) Day 1 at initial presentation showing vesiculopustular lesions with early punched‐out erosions, (B) Day 2 of admission with progression, (C) Day 5 of admission showing improvement, and (D) complete resolution at 2 weeks.

However, on the second day of admission, the patient showed clinical deterioration, with the development of pseudomembranous conjunctivitis and marked worsening of eyelid swelling (Figure 1B). Dermatology was consulted. Further clinical evaluation revealed that the morphology and distribution of the lesions—particularly their monomorphic appearance, punched‐out erosions, and involvement of areas previously affected by AD—were more consistent with EH. No bedside investigations such as Tzanck smear or microbiological studies were performed, and further virology tests including polymerase chain reaction (PCR) were not pursued due to practical and resource considerations, as well as parental preference. The diagnosis was revised to EH with ocular involvement based on the characteristic clinical features and disease progression.

Treatment was escalated to intravenous (IV) acyclovir at 10 mg/kg every 8 h, with additional IV cloxacillin (25 mg/kg every 6 h) for suspected secondary bacterial infection and topical ciprofloxacin eye drops for pseudomembranous conjunctivitis. The left eye also underwent daily rodding to remove the pseudomembranes. Despite this, the clinical condition further worsened and progressed to orbital cellulitis, evidenced clinically by severe conjunctival chemosis and radiologically by contrast‐enhanced computed tomography of the orbit, which demonstrated left preseptal soft tissue swelling with postseptal extension and medial extraconal fat stranding (Figure 2). The pediatric team was consulted, and antibiotic therapy was escalated to IV amoxicillin–clavulanate (30 mg/kg every 8 h) for broader coverage.

**FIGURE 2:**
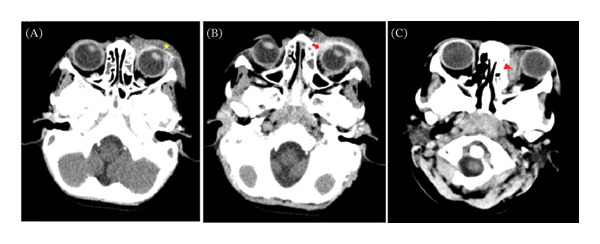
Axial contrast‐enhanced CT images at different orbital levels showing (A) left preseptal soft tissue swelling and (B) postseptal extension with (C) fat streakiness in the medial extraconal space (arrow).

Topical acyclovir 3% ointment was added on Day 4 of admission as adjunctive therapy at a frequency of five times daily, while IV acyclovir was continued. Following the addition of topical therapy, a steady clinical improvement was observed over the subsequent days (Figure 1C). The acyclovir ointment was continued for 1 week and tapered over another week. Complete resolution of cutaneous and orbital symptoms was achieved after 2 weeks, with no residual complications (Figure 1D).

## 3. Discussion

EH, also referred to as a type of Kaposi varicelliform eruption, is a cutaneous viral infection that typically manifests as a widespread vesicular skin eruption involving the face, head, and neck regions in patients with underlying AD. [1]. Children with AD have an increased risk of developing EH, with HSV Type 1 being the most frequently identified pathogen [2]. The exact pathophysiology of EH remains incompletely understood; however, it is believed to be related to immune dysregulation in AD, likely secondary to impaired interferon gamma (IFN‐γ)–mediated immunity [1]. A predominance of Th2‐mediated response further contributes to impaired skin barrier function and increased susceptibility to viral infection [1].

The diagnosis of EH is primarily clinical, based on the presence of monomorphic vesiculopustular or papular eruptions that evolve into characteristic “punched‐out” erosions, typically occurring on areas previously affected by AD [1, 2, 5]. Ocular involvement in EH is rare. Reported ophthalmic manifestations mainly involve the ocular surface, including conjunctivitis, keratoconjunctivitis, and keratitis, with or without dendritic or geographic ulceration [3, 4, 6]. Posterior segment involvement and progression to more severe intraorbital complications are exceedingly rare [1, 2].

Early suspicion and timely initiation of antiviral therapy remain critical in the management of EH, regardless of the site of involvement. When the diagnosis is uncertain, a Tzanck smear provides a rapid and cost‐effective bedside test, although it lacks specificity [1, 2, 5]. Additional diagnostic methods include vesicular fluid culture, viral serology, and histopathological examination [1, 2, 5]. Detection of HSV DNA by PCR and immunofluorescence can further support the diagnosis of EH; however, these methods are not always readily available or feasible in all clinical settings [1].

Systemic antiviral agents such as acyclovir are virostatic; they act by inhibiting viral replication and reducing disease severity, rather than exerting a direct virucidal effect [1]. Our case represents a severe form of EH with ocular involvement. The rapid progression to preseptal and orbital cellulitis was most likely related to secondary bacterial infection rather than direct intraorbital viral spread. While systemic bioavailability is higher with IV administration, topical acyclovir ointment provides targeted antiviral activity at the site of infection [1, 2, 6]. The improvement observed following the addition of topical acyclovir likely reflects the combined effect of systemic therapy and adjunctive topical treatment, along with the antibiotic rather than a direct effect of topical acyclovir alone.

Delayed or missed diagnosis in the early stage is not uncommon and does not necessarily reflect clinical oversight. Previous reports of pediatric EH have described similar diagnostic challenges [1, 5–7]. Prior to the appearance of characteristic lesions, EH may be misinterpreted as allergic reactions, contact dermatitis, or cellulitis, while early vesicular lesions may resemble impetigo or shingles [5]. EH lesions are typically more widespread, whereas HZO presents unilaterally along a single dermatome, and impetigo is characterized by small pustules with honey‐colored crusts [5]. Notably, the cutaneous manifestations of EH can be variable, especially in infants and young children, and may resemble conditions such as seborrheic dermatitis (“cradle cap”) or present with atypical clustered lesions and hemorrhagic crusts, contributing to diagnostic uncertainty [7, 8]. Most cases respond well to oral antiviral therapy with relatively low rates of severe complications and often do not require hospitalization [9, 10]. However, bacterial superinfection is a recognized complication that may increase disease severity and necessitate closer monitoring or inpatient management [2, 5, 6, 9, 10]. Although antibiotics are not routinely indicated, they are frequently used when secondary bacterial infection is suspected, as in our case, which justified escalation to IV therapy.

Effective comanagement involving dermatology, pediatrics, and ophthalmology is crucial in achieving successful outcomes for children with EH [2, 5, 6, 9]. The clinical improvement observed in this case following appropriate diagnosis and treatment underscores the importance of continuous reassessment and timely adjustment of management based on disease progression. Ultimately, this case highlights the complexities involved in diagnosing and managing pediatric EH. Early recognition and prompt antiviral therapy are crucial in preventing severe complications and optimizing clinical outcomes.

## Funding

The authors received no financial support for the study, authorship, and/or publication of this article.

## Ethics Statement

Ethical approval was not required for reporting an individual case in accordance with institutional policy. Written informed consent for publication of this case report and accompanying images was obtained from the patient’s parent. All efforts have been made to protect the patient’s identity.

## Conflicts of Interest

The authors declare no conflicts of interest.
